# Water corrosivity of polluted reservoir and hydropower sustainability

**DOI:** 10.1038/s41598-020-68026-x

**Published:** 2020-07-06

**Authors:** Sunardi Sunardi, Miranti Ariyani, Mochammad Agustian, Susanti Withaningsih, Parikesit Parikesit, Hafizan Juahir, Azimah Ismail, Oekan S. Abdoellah

**Affiliations:** 1 Center for the Environment and Sustainability Science (CESS), Universitas Padjadjaran, Jl. Sekeloa Selatan 1, Bandung, West Java Province 40132 Indonesia; 2 Research Unit for Clean Technology, Indonesian Institute of Sciences, Jl. Cisitu, Sangkuriang, Bandung, 40135 Indonesia; 3 BPWC – PT PJB (Cirata Reservoir Management Agency – PT Jawa Bali Powerplant), Indonesia, Jl. Raya Cirata, Cikalongwetan, Bandung Barat, West Java Province Indonesia; 4 Department of Biology, Faculty of Mathematics and Natural Science, Universitas Padjadjaran, Jl. Raya Bandung-Sumedang Km. 21 Jatinangor, Sumedang, West Java Province 45363 Indonesia; 5 Department of Anthropology, Faculty of Social and Political Science, Universitas Padjadjaran, Jl. Raya Bandung-Sumedang Km. 21 Jatinangor, Sumedang, West Java Province 45363 Indonesia; 6 East Coast Environmental Research Institute, Universiti Sultan Zainal Abidin, Gong Badak Campus, 21300 Kuala Terengganu, Terengganu Darul Iman Malaysia

**Keywords:** Environmental sciences, Limnology

## Abstract

Reservoirs play a strategic role in the context of sustainable energy supply. Unfortunately, the majority of the reservoirs are facing water-quality degradation due to complex pollutants originating from activities both in the catchment and inside the reservoir. This research was aimed at assessing the extent of the water degradation, in terms of corrosivity level, and at examining its impacts on hydropower capacity and operation. Water quality data (total dissolved solids, pH, calcium, bicarbonate, and temperature) were obtained from 20 sampling stations in the Cirata Reservoir from 2007 to 2016. The results show that the river water is already corrosive (Langelier Saturation Index, LSI = − 0.21 to − 1.08), and, the corrosiveness becoming greater when entering the reservoir (LSI = − 0.52 to − 1.49). The water corrosivity has caused damage to the hydro-mechanical equipment and lowering production capacity. The external environment of the catchment hosts complex human activities, such as agriculture, land conversion, urban and industrial discharge, which have all played a major role in the water corrosiveness. Meanwhile, the internal environment, such as floating net cage aquaculture, has intensified the problem. As the water corrosiveness has increased, the maintenance of the hydro-mechanical facilities has also increased. Strategies must be applied as current conditions are certainly a threat to the sustainability of the hydropower operation and, hence, the energy supply.

## Introduction

Currently, water quality has become increasingly more important in the context of reservoir sustainability and pristine management. The increasing human population, industrialization, and other anthropogenic activities, such as agriculture and domestic activities, have caused changes in the physical, chemical, and biological conditions of reservoir water^1^. Corrosivity is one of the qualitative measures of water quality that indicates the presence of contaminants^2^ and is associated with the continuity of hydropower facilities. Corrosion is related to the acidity of the hydroenvironment that determines the sustainability of energy provision because corrosion leads to costly maintenance of hydro-mechanical equipment, and hence the sustainability of power production^3,4^.

Corrosion is a physicochemical interaction process between metal and its surroundings^5,6^. Several indicators can be used to measure the corrosivity of water; among others is the Langelier Saturation Index (LSI)^7,8^. The LSI has been employed in the corrosivity analysis of drinking water^5,6,9,10^, underground water^2^, river water^11^, and water from treatment plants^12^. High levels of pollution, caused by various activities, causes hydro-mechanical equipment to degrade. Kumar et al.^13^ reported that the Thatipudi reservoir water in Andhara Pradesh has experienced corrosiveness, which has also potentially damaged the water distribution system.

Cirata is one of the cascade reservoirs located on the Citarum River, which plays a strategic role in supporting development in Indonesia. The reservoir was established in 1987, with the main purpose being energy provision through hydropower plant construction, to provide 1,008 GWh electricity for Java and Bali^14^. Its role has been expanded, however, to include water supply to cities, irrigation, flood control, tourism, plant and animal conservation, and aquaculture. The continuity of Cirata hydropower substantially depends on the condition of the Citarum watershed, in which various socioeconomic activities have spread from the cities of Bandung, Soreang, Majalaya, Banjaran, Cimahi, etc.

Apparently, the intensive socioeconomic activities, and tremendous land-use changes in the catchment, will contribute to the increasing sedimentation in the reservoir. In the Master Plan document, it was reported that, in 2007, the actual sedimentation rate in Cirata Reservoir reached up to 3.96 mm/year, greatly exceeding the estimated sedimentation rate of 1.2 mm/year^15^. Other pollutant sources, such as domestic, industrial, and agricultural activities, and leachate generated from landfill, may contribute to the accumulation of organic matter on the bottom of the reservoir. The decay of organic matter in the sediments results in highly toxic and corrosive substances in the water. The water pollution is exacerbated by the presence of a large population of floating net cages, which reach up to 68,000 units^16^. This figure exceeds the number set by the Government of West Java Province through Governor’s Decree No. 41 2002, which allowed only about 20,000 units. Moreover, the majority of the farmers who are engaged in aquaculture use food pellets in an inefficient manner, causing higher residual sedimentation in the reservoir. Uneaten pellets from the floating net cages could possibly be the main factor that is contributing to the water quality degradation, through significant organic decomposition.

This work aims to assess the impacts of the water quality degradation on hydropower sustainability in Cirata Reservoir. Referring to the fact that hydro-mechanical equipment may be degraded by corrosive water, the capacity of the hydropower plant may be decreased. Cirata Reservoir is experiencing serious water pollution; assessing the corrosiveness of the reservoir water, and evaluating its impact on hydro-mechanical equipment, will provide much-needed insight into how water quality may determine hydropower sustainability.

## Results

### Trend of reservoir water quality

**Figure 1:**
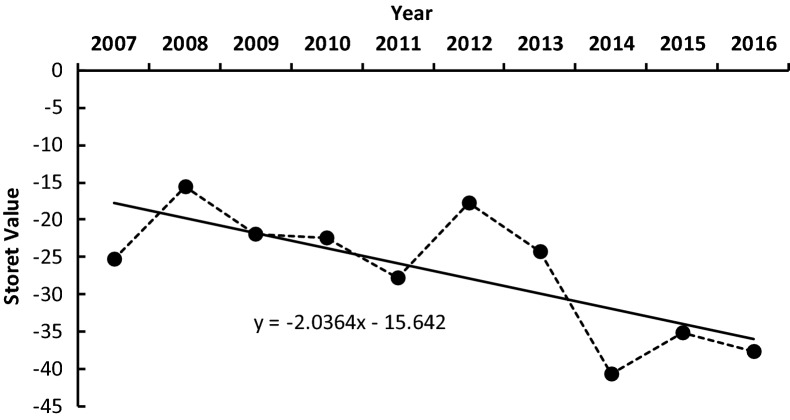
Trend in the water quality of Cirata Reservoir (indicated by Storet Index values) from 2007 to 2016.

Regular monitoring activities in Cirata Reservoir found that the water quality had been degrading for the last 10 years (Fig. 1). Refering to the national regulations, the Storet Index showed that some of the physicochemical parameters did not meet the recommended standards. Based on our estimations, the Storet Index values ranged between − 15 and − 40, with an average of − 26. This indicates that the quality of the water in the Cirata Reservoir was medium to highly polluted, and that this has worsened over time.

### Corrosivity of the tributary and reservoir water

Our assessment of the reservoir water revealed that Cirata, in general, has experienced substantial water corrosivity over the past 10 years (Table 1).

**Table 1:** Water corrosivity of river tributaries, and sampling locations in the reservoir.

No	Station	LSI	Note
**Tributaries**			
1	Citarum-Cimeta encounter area (Station 7)	− 0.50	Corrosive
2	Cimeta river-mouth (Station 8)	− 0.37	Corrosive
3	Cikundul river-mouth (Station 10)	− 0.82	Corrosive
4	Cilaku river-mouth (Station 12)	− 0.64	Corrosive
5	Cisokan River (Station 13)	− 0.61	Corrosive
6	Citarum River, before Cimeta encounter (Station 14)	− 1.08	Corrosive
7	Cimeta River (Station 15)	− 0.32	Corrosive
8	Cipicung River (Station 16)	0.21	Scale-forming
9	Cicendo river-mouth (Station 17)	− 0.41	Corrosive
**Reservoir**			
1	Bandung Barat district zone (Station 1)	− 0.77	Corrosive
2	Trashboom area (Station 2)	− 1.32	Corrosive
3	Border of aquaculture-free area (Station 3)	− 1.14	Corrosive
4	Purwakarta district zone (Station 4)	− 0.52	Corrosive
5	Cisokan river-mouth (Station 5)	− 0.59	Corrosive
6	Citarum river-mouth (Station 6)	− 0.82	Corrosive
7	Dam site (Station 9)	− 0.92	Corrosive
8	Cibalagung river-mouth (Station 11)	− 1.28	Corrosive
9	Cilangkap river mouth (Station 18)	− 1.49	Corrosive
10	Midpoint zone of Purwakarta (Station 19)	− 1.18	Corrosive
11	Midpoint zone of Cianjur (Station 20)	− 0.61	Corrosive

**Figure 2:**
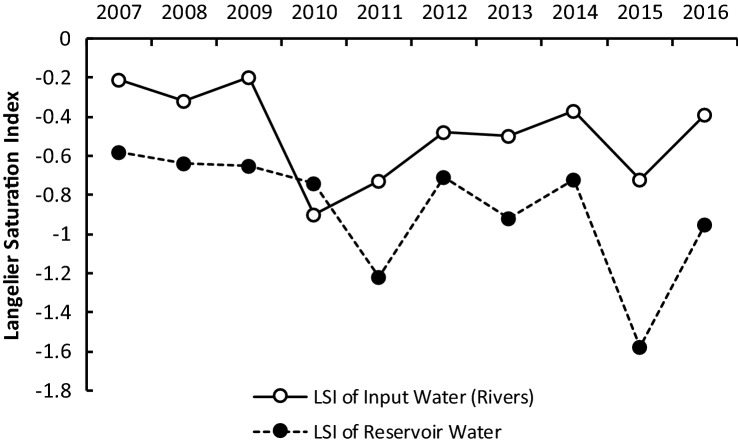
Water corrosivity level of the Cirata Reservoir and its tributaries, 2007 to 2016.

Both the tributaries and reservoir water have been confirmed as having negative LSI values, indicating the potential for corrosion (Fig. 2).

### Reservoir water corrosivity between floating and non-floating cage areas

The compelling scenery of the Cirata Reservoir is the abundant population of floating net cages. According to Table 2, the water in the area of dense floating net cages tends to be more corrosive than in the area where these are absent. Statistical analysis, however, shows that the difference between water corrosiveness in the caged and empty areas was not significant (T-test, *p* > 0.05, 0.584 > 0.05).

**Table 2:** Corrosivity levels of water in caged and empty areas, Cirata Reservoir.

No	Station	Corrosivity index (LSI)	Note
**Zone of floating net cages**			
1	Bandung Barat district zone (Station 1)	− 1.49	Corrosive
2	Midpoint zone of Purwakarta (Station 19)	− 1.18	Corrosive
3	Midpoint zone of Cianjur (Station 20)	− 0.61	Corrosive
Average		− 1.09	Corrosive
**Zone of no floating net cages**			
1	Bandung Barat district zone (Station 4)	− 1.23	Corrosive
2	Cisokan river-mouth (Station 5)	− 0.52	Corrosive
3	Citarum river-mouth (Station 6)	− 0.62	Corrosive
4	Cibalagung river-mouth (Station 11)	− 0.92	Corrosive
5	Cilangkap river-mouth (Station 18)	− 1.34	Corrosive
Average		− 0.92	Corrosive

### Hydro-mechanical degradation caused by corrosive water

The data showed that the frequency of maintenance (overhaul, predictive and corrective maintenance) was higher when the water corrosiveness increased in the period 2009 to 2013 (Table 3). It was proven that the degradation of water quality caused damage to the hydromechanical equipment, and thus disrupted the hydropower capacity and operation.

**Table 3:** Frequency of maintenance of the hydro-mechanical equipment in the Cirata hydropower station.

Type of maintenance	Frequency				
	2009	2010	2011	2012	2013
Predictive maintenance	9	10	29	48	63
Overhaul	2	3	6	6	5
Corrective maintenance	112	146	234	200	140
Total	123	159	269	254	208

## Discussion

The pollution of the Cirata water has threatened the operation of the hydropower plant, as well as affecting other activities, such as aquaculture, tourism, monitoring activities, etc. The common problems apparent in the Cirata Reservoir due to water pollution are hydro-mechanical corrosion, fish kill in the floating net cage aquaculture, excessive growth of algae and water hyacinth, and water pollution by heavy metals^17–20^. As a eutrophic water, the environmental conditions, such as high nutrients, have triggered the microscopic algae growing excessively in the Cirata Reservoir. Similar condition has been found in deep drinking water reservoir, the Jinpen Reservoir in China^21^. Olivia et al.^22^ indicated that microorganism, such as microalgae and bacteria, in the water can induce microbiologically influenced corrosion that leads to degradation of the concrete structures.

Activities in the catchment seem to have had an influence on the corrosiveness of the input water, while the activities within the reservoir have intensified the water corrosiveness. Ekholm et al.^23^ regarded that the catchment charasteristics had affected the concentration of nutrients and total suspended solids in the river system. The average water corrosiveness in the reservoir was significantly higher, compared to that in the tributaries (T-test, *p* < 0.05, 0.04 < 0.05).

The contribution of activities in the catchment to the water corrosiveness seems to be greater than those in the reservoir area. The catchments host complex activities, and contain sources, that cause the input water to be corrosive. The Institute of Ecology, Universitas Padjadjaran (2010) reported that the catchments had contributed up to 675,768 ton/year of particulate matter from suspended and dissolved solids alone; however, when the dissolved and deposited material from the rivers is considered, the total amount of material causing the corrosiveness is much higher. Garno^24^ stated that an amount of 145,334 ton/year of organic matter came from the fish cages in the reservoir, which is partially deposited, suspended or dissolved in the water.

Based on the scientific evaluation of each input into the water, i.e., from the tributaries, there is an indication that these waters were already corrosive, which increases the level of corrosiveness when the water enters the reservoir (Table 1). Most of the sampling stations showed that the water corrosivity in the catchment area was high. Due to natural geographical features, the water changed from flowing in the rivers to stagnant in the reservoir, potentially causing different precipitation rates among chemical compounds. Further, this indicates that eutrophication generates a high potential for water corrosivity in the reservoir.

Considering the input water, the contribution from the nine rivers flowing into the Cirata Reservoir has obviously worsened the water quality. Various activities in the catchment area, such as agriculture, residential settlement, and commercial, industrial and municipal wastewater discharge, to some extent have contributed to the reservoir water degradation. Out of the nine rivers, the Citarum, Cikundul, Cilaku, and Cisokan rivers contributed the most to the water quality degradation. These four rivers run through the most densely-populated parts of the catchment, where human activities are most concentrated. The Citarum river itself is the longest river in the West Java Province, crossing the Greater Bandung, the capital of the province.

The main feature related to the corrosivity of the four rivers has been confirmed as high total dissolved solid. The concentration of total dissolved solid indicates the amount of material dissolved in the water, including both organic and inorganic ions, such as chloride, nitrate, sulfate, phosphate, sodium, magnesium, calcium, iron, and aluminium Kumar et al.^13^. The type of material contained in the total dissolved solid affect the properties of water corrosivity. When it contains more corrosive ions, such as chloride, the water becomes corrosive, or has a strong tendency to form carat^13^. Previous studies have shown that the upper Citarum basin has experienced serious environmental degradation, due to massive conversion of land for settlement and agriculture^25^, pesticide and fertilizer pollution from intensive horticulture and paddy fields^26^, and domestic waste from urban centres^27^.

The floating cages are common in the reservoir, hosting tilapia, common carp, catfish, and pangasius. The number of cages is countinuously increasing, and, according to the last census, the population of the cages had reached 68,000 units in 2013^16^. The floating net cage activities significantly deleteriously impact water corrosivity, due to the excessive amounts of residual feed derived from it. Fish food contains organic ingredients, nutrients, and other chemical elements that increase the pollution load in the reservoir^28^. Garno^24^ estimated that, during the last five years, the floating net cages in the Cirata Reservoir had generated as much as 145,334 tons/year of organic waste, containing 6,611.8 tons nitrogen and 1,041.4 tons phosporus. The implication concerning water corrosiveness is clear; the level of corrosivity in dense floating net cages are higher than that in the area where the floating net cage are absent (see Table 2).

Negative effects of the corrosive water in the Cirata Reservoir were noticable, particularly in hydro-mechanical equipment, such as turbines, generator mechanical sets, air coolers, brake valves, piping, etc. The problems of corrosion were more severe for metal instruments that had direct contact with the reservoir water. The maintenance records indicated that a greater effort of maintenance on the hydro-mechanical equipment corresponded to the worsening water quality.

Organic matter entering the water is another issue related to water corrosivity. This originates mainly from settlements, industry and the floating net cages. The presence of organic matter in the water column, as represented by the dissolved and suspended solids content, contributed to the increased maintenance of the hydro-mechanical equipment through corrosion and cavitation, due to the nucleation of the turbine^29^. Cavitation is a process whereby water bubbles form due to pressure differences, and where the rupture of the bubbles potentially corrodes the turbine^30^. Gregorc et al.^31^ stated that the presence of particles in the water can increase cavitation due to increasing pressure differences in water-containing particles. Thus, the higher the solid content of water, the greater the possibility of corrosion^32^.

Aside from organic matter, the corrosiveness of the water in the reservoir area is also triggered by dissolved and suspended solids from activities such as inappropriate agricultural practices and land conversion. If particulate matter does not settle, it increases turbidity in the water column for a longer amount of time, resulting in greater corrosion of the hydro-mechanical equipment^29^. The utilization of synthetic fertilizer, such as NPK, urea, TSP, and potassium chloride, also generates chemical residues, such as ammonia, phosphate, sulfate, and chloride, which are corrosive^33^. Ammonia is a major source of nitrogen in synthetic fertilizers, produced through a reaction of pure nitrogen gas and hydrogen forming ammonium nitrate, which is highly corrosive^34^. Liao et al.^35^ stated that ammonium, nitrate, nitrite, and chloride ions may lead to localized corrosion, which will eventually damage metallic equipment. Kinouchi et al.^36^ regarded phosphate compounds generated from fertilizers in agricultural activities to be a compound that would be easily leached into rivers. According to Biglari et al.^2^ phosphate is one of the chemical compounds that contributes to water corrosiveness properties.

Water corrosivity in the Cirata Reservoir is a real threat to the sustainability of the hydropower operations, and hence to the energy supply of Java and Bali; however, is has been shown that the catchments have been a greater influence on water corrosivenes than the activities in the reservoir. Several strategies may be applied to cope with the corrosivity problem, such as controlling pollution from the catchments, and adopting a more eco-friendly aquaculture. The urban centers may also need to provide effective waste water treatment for domestic discharge; while agriculture could use pesticides and fertilizer more efficiently. Sustainable agriculture could also be the best option for future agricultural sector development. In the reservoir area, a reduction of floating net cages, alternative feeding practices, such as automated feeding, and provision of more digestable pellets, should be considered.

## Methods

### Research site

**Figure 3:**
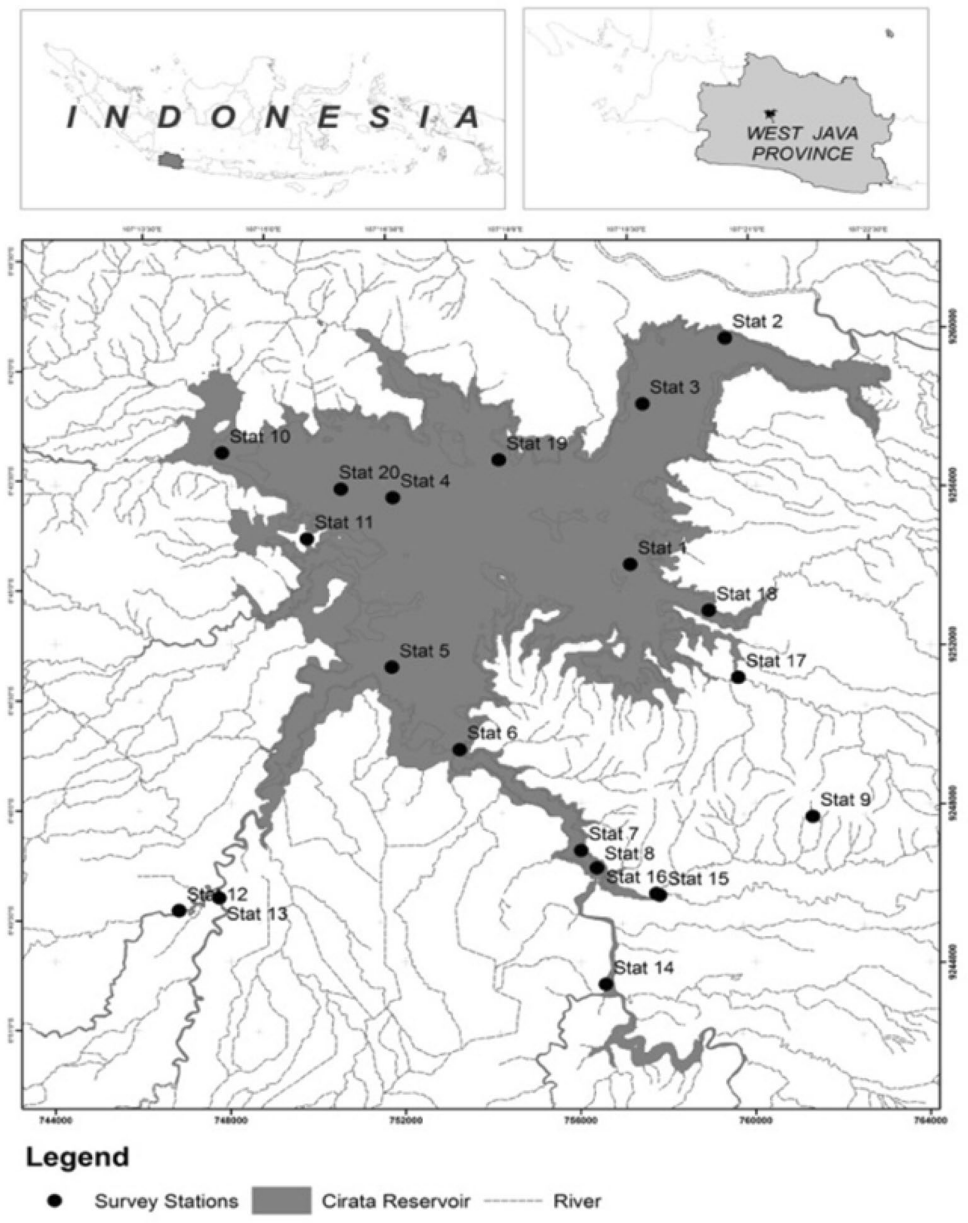
Water sampling points in the Cirata Reservoir. Note: (1) Bandung Barat district zone, (2) trashboom area, (3) border of aquaculture-free area, (4) Purakarta Barat district zone, (5) Cisokan river-mouth, (6) Citarum river-mouth, (7) Citarum-Cimeta encounter area, (8) Cimeta river-mouth, (9) dam area, (10) Cikundul river-mouth, (11) Cibalagung river-mouth, (12) Cilaku river-mouth, (13) Cisokan River, (14) Citarum River (before Cimeta encounter), (15) Cimeta River, (16) Cipicung River, (17) Cicendo river-mouth, (18) Cilangkap river-mouth, (19) midpoint zone of Purwakarta, and (20) midpoint zone of Cianjur.

The study was conducted in the Cirata Reservoir, located in West Java Province, from 2007 to 2016. The reservoir is one of the cascade reservoirs in the Citarum River Basin with a very strategic role in development at both the local and national levels. The inundation of the Cirata Reservoir was started in September, 1987, with covered an area of 62 Km^2^. The priority goal of the reservoir development was to provide support for the national electricity need through establishment of a hydropower with capacity of 1,008 MW. However, as an open fostered ecosystem the Cirata Reservoir in fact has multiple functions such as to support aquaculture activities, irrigation, transportation and tourism. The sampling points were placed in both the inundation area, i.e., the reservoir, and the river-mouths where they flow into the reservoir (see Fig. 3); water samples were taken from 20 sampling points.

### Research procedure

This work was divided into two stages: (1) laboratory analysis of water samples; and (2) assessment of water corrosivity levels. Sampling of water from the reservoir was conducted quarterly, i.e., in February, May, August and October, from 2007 until 2016. The water samples were taken at a depth of 0.2 m from the surface, using a water sampler. The collection technique refers to SNI 03-7016-2004, regarding Sampling Procedures for Water Quality Monitoring of Rivers and Lakes, while the physicochemical characteritics of total dissolved solid, pH (acidity), calcium, bicarbonate, and temperature, were measured based on APHA AWWA WCPF^37^. The state of the water pollution is expressed as the Storet Index.

The data obtained were used to assess the level of water corrosivity, expressed in LSI^7,8^. When the LSI value was negative, it showed that the water had limited scale-forming potential, but tended to be corrosive. Conversely, when the LSI was positive, the water had a tendency to form scale. As the value increased, the scaling potential increased in tandem^13^. The formulae used were:

1 $$ LSI = pH - pHs $$

2 $$ pHs = \left( {9.3 + A + B} \right) - \left( {C + D} \right) $$

where the LSI indicates the driving force for scale formation and growth, in terms of pH as a master variable. pH is actual acidity, and pHs is the saturation acidity. pHs is calculated by the formulation of A, B, C, and D, as presented below:

3 $$ A = \frac{{(\log \left[ {TDS} \right] - 1)}}{10} $$

4 $$ B = { } - 13.12{ } \times \log \left( {{^\circ }{\text{C}} + 237} \right) + 34.55 $$

5 $$ C = \log { }\left[ {Ca^{2 + } as\,CaCO_{3} } \right] - 0.4 $$

6 $$ D = \log\,[alkalinity\,\,as\,\,CaCO_{3} ] $$

where the estimation of A, B, C, and D variables use alkalinity (mg/L as CaCO_3_), calcium hardness (mg/L Ca^2+^ as CaCO_3_), TDS (mg/L), and the temperature of the water (°C) as inputs.

The classification of water corrosivity levels of the LSI is presented in Table 4.

**Table 4:** Langelier saturation Index classification. *Sources*: Langelier^7^ and Carrier^8^.

No.	Value	Category
1	LSI > 0	Water is supersaturated and tends to precipitate a scale layer of CaCO_3_ forming scale
2	LSI = 0	Water is saturated (in equilibrium) with CaCO_3_ and will neither be strongly corrosive or scale forming
3	LSI < 0	Water is undersaturated and tends to dissolve solid CaCO_3_, and tend to be corrosive

To obtain information on the hydro-mechanical damage, maintenance data were collected from the relevant sections of the hydropower company, PT PJB. The data were accessible only for the years 2009 until 2013.

### Data analysis

To see if there were any differences in the water corrosiveness between the river and the reservoir water, and between the floating net cage and empty areas, the T-test was employed. Statistical analysis was performed using version R-3.6.1 of the ‘*R*’ software environment for Microsoft Windows.

## Conclusions

Water corrosiveness in the Cirata Reservoir has apparently threatened the continuity of hydropower operations, and hence the energy supply. In the reservoir, corrosive water has noticeably caused damage to the hydro-mechanical equipment, and increased the frequency of maintenance. The catchments, as the focus of complex human activities, have contributed the greatest influence to the water corrosiveness, while the activities and limnological processes in the reservoir have played a role in intensifying the corrosiveness of the reservoir water. Several strategies can be taken to cope with the problem, with measures to restrict harmful activities around the catchments to be made a priority.
